# Novel Role of Src in Priming Pyk2 Phosphorylation

**DOI:** 10.1371/journal.pone.0149231

**Published:** 2016-02-11

**Authors:** Ming Zhao, Darren Finlay, Irina Zharkikh, Kristiina Vuori

**Affiliations:** Cancer Center, Sanford Burnham Prebys Medical Discovery Institute, 10901 N. Torrey Pines Road, La Jolla, California, United States of America; Hungarian Academy of Sciences, HUNGARY

## Abstract

Proline-rich tyrosine kinase 2 (Pyk2) is a member of the focal adhesion kinase (FAK) family of non-receptor tyrosine kinases and plays an important role in diverse cellular events downstream of the integrin-family of receptors, including cell migration, proliferation and survival. Here, we have identified a novel role for Src kinase in priming Pyk2 phosphorylation and subsequent activation upon cell attachment on the integrin-ligand fibronectin. By using complementary methods, we show that Src activity is indispensable for the initial Pyk2 phosphorylation on the Y402 site observed in response to cell attachment. In contrast, the initial fibronectin-induced autophosphorylation of FAK in the homologous Y397 site occurs in a Src-independent manner. We demonstrate that the SH2-domain of Src is required for Src binding to Pyk2 and for Pyk2 phosphorylation at sites Y402 and Y579. Moreover, Y402 phosphorylation is a prerequisite for the subsequent Y579 phosphorylation. While this initial phosphorylation of Pyk2 by Src is independent of Pyk2 kinase activity, subsequent autophosphorylation of Pyk2 *in trans* is required for full Pyk2 phosphorylation and activation. Collectively, our studies reveal a novel function of Src in priming Pyk2 (but not FAK) phosphorylation and subsequent activation downstream of integrins, and shed light on the signaling events that regulate the function of Pyk2.

## Introduction

Upon cell adhesion to extracellular matrix, the integrin-family of transmembrane receptors are clustered at sites termed focal adhesions and activate various intracellular signaling pathways. Focal adhesion kinase (FAK) family members are important downstream mediators of integrin signaling in events such as cell proliferation, survival, motility and invasion, and are therefore considered as plausible drug targets in various disease processes, such as inflammation and cancer [1,2].

Proline-rich tyrosine kinase 2 (Pyk2), also known as RAFTK and CAK-β, is a non-receptor tyrosine kinase that shares related structure and sequence similarity with FAK [1,3,4]. Both of these kinases comprise an N-terminal FERM domain, a central kinase domain and a focal adhesion targeting (FAT) domain towards the C-terminus. They share roughly 45% amino acid sequence identity and 65% similarity [1,4,5]. While FAK is ubiquitously expressed, Pyk2 expression is mainly restricted to the central nervous system and hematopoietic cells [1]. Accordingly, Pyk2 has been shown to play a critical role in lymphocyte and macrophage migration [6,7]. Protein levels of Pyk2 are often upregulated in glioma cells [8], and inhibition of Pyk2 blocks glioma cell migration, implicating Pyk2 in glioma pathogenesis. Interestingly, Pyk2 protein levels are elevated in FAK^-/-^ mouse embryonic fibroblasts (MEFs) and Pyk2 can compensate for some but not all FAK functions in these cells [9,10]. Indeed, dual inhibition of FAK and Pyk2 may be needed as a potential therapeutic strategy in various disease settings that these kinases have been implicated in [11,12].

The underlying mechanism of FAK activation has been extensively studied. It has been shown that when FAK is inactive, the FAK FERM domain associates with and inhibits the FAK kinase domain, and deletion of the FERM domain promotes FAK activity [13]. Upon recruitment of FAK to ligand-bound integrins, a conformational change in FAK results in the release of the FERM domain binding, allowing autophosphorylation of tyrosine-397 (Y397) [14]. This is followed by binding of Src to phosphorylated Y397 of FAK [15,16] and phosphorylation of the FAK activation loop at residues Y576 and Y577 by Src. These phosphorylation events result in full catalytic activation of FAK [17,18,19].

Given the similarity in amino acid sequence and structure between FAK and Pyk2, and given that Pyk2 contains sites Y402 and Y579/580 that are considered equivalent to sites Y397 and Y576/577 in FAK, it was expected that Pyk2 would be similarly regulated. Several differences have been observed, however. For example, while the FERM domain of Pyk2 inhibits Pyk2 activity, it appears to exert its effects without interacting with the Pyk2 kinase domain [20]. Also, Pyk2 has been shown to dimerize when overexpressed and to undergo autophosphorylation *in trans* in a manner that appears to be independent of Src [21]. Further, Riggs *et al*. have reported that oligomerization of Pyk2 is important for Pyk2 activity and that the FERM domain plays an important role in this process [22]. In support of this, Kohno *et al*. have demonstrated that Ca2+/calmodulin binds to the Pyk2 FERM-domain, which results in Pyk2 activation through homodimer formation and transphosphorylation [23].

In this study, we show that Src activity is indispensable for the initial Pyk2 phosphorylation at site Y402 upon cell attachment. In contrast, and as shown before by others, fibronectin-induced autophosphorylation of FAK at the corresponding Y397 site occurs in a Src-independent manner. We further demonstrate that the Src SH2 domain is required for Src-Pyk2 interaction, and thus plays key role in Src phosphorylation of Pyk2. While this initial phosphorylation of Pyk2 by Src is independent of Pyk2 kinase activity, our results suggest that subsequent autophosphorylation of Pyk2 *in trans* is required for full Pyk2 phosphorylation and activation. Collectively, our studies uncover a novel role for Src in priming Pyk2 phosphorylation and activation, and shed light on the signaling events that regulate the function of Pyk2.

## Materials and Methods

### Materials

Anti-phospho-Pyk2 (Y402), anti-Pyk2, anti-Src and anti-α-tubulin antibodies were from Cell Signaling Technologies (Beverly, MA); anti-phospho-Pyk2 (Y579) was from EMD Millipore (Billerica, MA); anti-phospho-FAK (Y397) and anti-FAK monoclonal antibodies used in immunoblot analysis were from Transduction Laboratories (Lexington, KY); anti-phospho-FAK (Y576) was purchased from Invitrogen (Carlsbad, CA); and anti-β-actin antibody was from Sigma (St. Louis, MO). Fibronectin was from Invitrogen (Carlsbad, CA). Src inhibitor SU11333 [24] was provided by Dr. Sara Courtneidge (Sanford Burnham Prebys Medical Discovery Institute), PP2 was purchased from EMD Millipore.

### DNA constructs

Myc-tagged wild-type and mutant Pyk2 plasmids were from Dr. Wencheng Xiong (Georgia Regents University), and were subcloned to pEGFP-C1 (Invitrogen) to create GFP-Pyk2 constructs. Wild-type Src and kinase-dead Src (K295N) constructs were obtained from Dr. Sara Courtneidge, and Src mutants Y416F, ΔSH2 and ΔSH3 were provided by Dr. Matthias Hentze (EMBL). Src SH2 and SH3 defective mutants R175L and W118K and the double mutant R175L/W118K were created by using the Quick Change Mutagenesis Kit (Agilent Technologies, Santa Clara, CA). Pyk2-WT (amino acids 1–408) and Pyk2-Y402F (amino acids 1–408) were subcloned to pGEX-4T-1 as BamHI-XhoI fragments (GE Healthcare Life Sciences, Pittsburgh, PA) to generate GST-Pyk2 constructs.

### Cell culture and transfection

HeLa cells, 293T cells, SYF fibroblasts derived from mouse embryos lacking three Src-family kinases (Src, Fyn, and Yes) and Src^++^ MEF cells (SYF cells with Src-expression reconstituted) [25] were from ATCC, and were cultured in DMEM plus 10% fetal bovine serum. Cells were transiently transfected using Fugene HD reagent (Roche, Indianapolis, IN) as indicated, and 24–48 hours later, the transfected cells were subjected to the various assays described here.

### Immunoblotting

Cell monolayers were serum-starved overnight, trypsinized and treated as described in the figure legends. Cells were then lysed in modified RIPA lysis buffer (25 mM Tris-HCl, pH7.4, 10% glycerol, 0.2% Triton X-100, 150 mM NaCl, 1 mM EDTA, 1 mM EGTA) containing a protease inhibitor cocktail (Roche Applied Science, Indianapolis, IN), and clarified by centrifugation. Protein concentrations of each sample were determined by Bradford assay. Equal amounts of protein were resolved by SDS-PAGE, transferred to a nitrocellulose membrane, blocked with 3% dry milk (Bio-Rad, Hercules, CA) in TBS-Tween-20 and exposed to specific primary antibodies as described for each experiment. Antibody binding was detected using horseradish peroxidase (HRP)-conjugated goat anti-rabbit or anti-mouse secondary antibodies (Sigma) and enhanced chemiluminescence (GE Healthcare Life Sciences, Pittsburgh, PA). Tubulin or β-actin served as internal loading controls.

### Immunoprecipitation

SYF cells transfected as described in the figure legends were lysed in modified RIPA buffer and the lysates were clarified by centrifugation. After pre-cleared with protein A beads (GE Healthcare Life Sciences), the supernatants were incubated in anti-Src antibody (Cell Signaling) with gentle rocking at 4°C, followed by capture of the immunocomplexes with protein A beads for 1 h. The immunoprecipitates were thoroughly washed with lysis buffer to remove nonspecifically bound proteins, and then analyzed by immunoblotting as described above.

### *In vitro* phosphorylation of Pyk2

GST fusion proteins of Pyk2-WT and Pyk-Y402F were expressed from pGEX4T plasmids in BL21 DE3 *E*. *coli* strain and purified by using GST Spin Trap column (GE Healthcare Life Sciences) following manufacturer’s instructions. Purified GST-Pyk2 WT or Y402F were incubated with recombinant Src kinase (Millipore, Billerica, MA), and phosphorylated Pyk2 was examined by immunoblotting using anti-Pyk2-pY402 antibody.

## Results

### Src activity is required for adhesion-mediated Pyk2 phosphorylation

**Fig 1:**
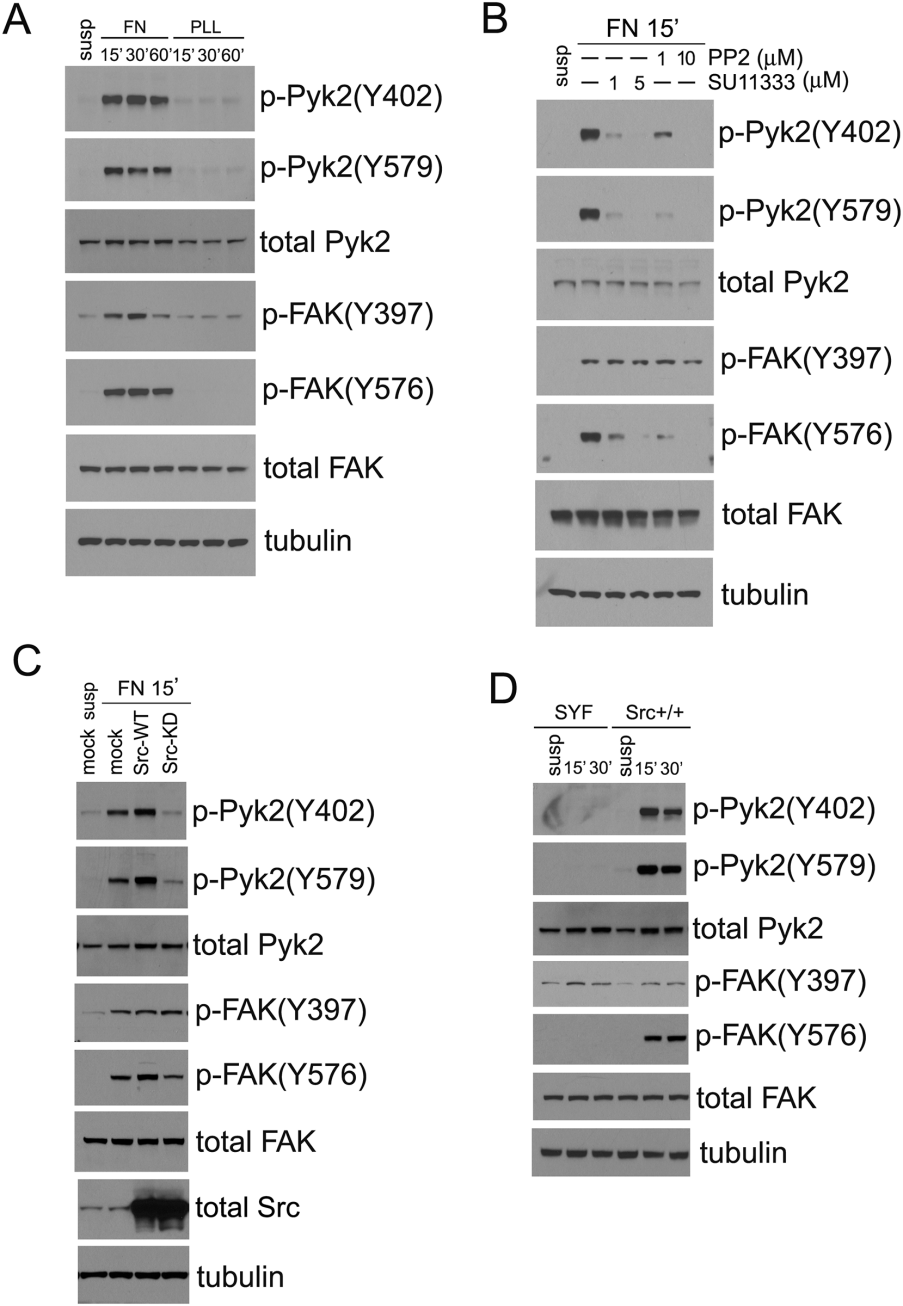
Src activity is required for Pyk2 phosphorylation in cells plated on fibronectin. A, serum-starved HeLa cells were detached and kept in suspension in DMEM containing 0.5% BSA for 1 hr. Cells were then seeded on fibronectin- or poly-l-lysine (PLL)-coated dishes, and allowed to attach for the indicated times. Phosphorylation of Pyk2 and FAK was examined by immunoblot, as indicated. B, HeLa cells were kept in suspension with or without indicated Src inhibitors for 1 hr before the attachment to fibronectin-coated dishes for 15 min, and phosphorylation of Pyk2 and FAK was examined as above. C, HeLa cells transfected with mock, wild-type Src (Src-WT) or kinase-dead mutant (Src-KD) were kept in suspension for 1 hr then were attached to fibronectin-coated dishes for 15 min, and the phosphorylation of Pyk2 and FAK was examined by immunoblot as indicated. D, serum-starved SYF and Src^++^ MEFs were kept in suspension for 1 hr, then were attached to fibronectin-coated dishes for 15 or 30 min as indicated, and the phosphorylation of Pyk2 and FAK was examined as in above.

Previously, Src has been shown to interact with and phosphorylate FAK and Pyk2 upon integrin ligation by extracellular matrix proteins [26,27]. Here we show that, as expected, attachment of cells to fibronectin elicited robust phosphorylation at both Y402 and Y579 sites of Pyk2 (Fig 1A). FAK phosphorylation was examined in comparison and FAK similarly demonstrated phosphorylation at the homologous Y397 and Y576 sites upon cell adhesion to fibronectin but not to the non-integrin ligand poly-L-lysine (Fig 1A).

To examine the role of Src in adhesion-mediated phosphorylation of Pyk2 and FAK, two Src kinase inhibitors, SU11333 and PP2, were used. We observed that fibronectin-mediated Pyk2 phosphorylation was significantly reduced at both the Y402 and Y579 sites by these inhibitors (Fig 1B). In contrast, FAK-Y397 phosphorylation was largely unaffected in the presence of the Src inhibitors (Fig 1B). Phosphorylation of FAK-Y576, a well-known Src phosphorylation site [28], however, was effectively inhibited by the Src inhibitors. Thus, these results suggest that fibronectin-induced tyrosine phosphorylation of Pyk2 and FAK at Y402 and Y397 sites, respectively, are differentially regulated by Src. Src is required for Pyk2-Y402 phosphorylation upon cell adhesion, whereas FAK-Y397 phosphorylation occurs independent of Src, presumably due to autophosphorylation [15]. Both Pyk2-Y579 and FAK-Y576 phosphorylation events were found to be Src-dependent (Fig 1B).

In support of these findings, we observed that transient over-expression of wild-type Src (Src-WT) significantly promoted fibronectin-induced Pyk2-Y402 phosphorylation, but it had no effect on FAK-Y397 phosphorylation (Fig 1C). A kinase-dead form of Src (Src-KD) in turn inhibited Pyk2-Y402 phosphorylation in a dominant-negative manner upon cell adhesion on fibronectin, but it failed to inhibit FAK-Y397 phosphorylation (Fig 1C). Consistent with the chemical inhibition of Src (Fig 1B), Src-WT enhanced, and Src-KD inhibited phosphorylation of both Pyk2-Y579 and FAK-Y576 (Fig 1C).

To complement these results obtained in HeLa cells, we found that in cells that are genetically deficient in the Src family kinases Src, Yes and Fyn (SYF cells) [25], fibronectin-mediated Pyk2-Y402 phosphorylation was completely absent, while FAK-Y397 phosphorylation was comparable to that in SYF cells with Src re-expressed (Src^++^) (Fig 1D). As expected, phosphorylation of both Pyk2-Y579 and FAK-Y576 was observed only in adherent Src^++^ cells, but not in SYF cells (Fig 1D). All these data demonstrate that Src activity is required for fibronectin-induced phosphorylation of Pyk2 at Y402, but not for FAK phosphorylation at the corresponding Y397 site. Phosphorylation of the activation loop Y579 and Y576 in Pyk2 and FAK, respectively, however, appear to be Src-dependent, as reported previously [26,27].

### Src phosphorylation of Pyk2 is largely dependent of Src SH2-domain

In addition to the tyrosine kinase catalytic domain, Src possesses N-terminal regulatory regions termed SH2- and SH3-domain. These modular domains mediate protein-protein interactions that are important in signal transduction [29,30,31]. To examine the putative roles of these domains in Pyk2 phosphorylation, the following Src mutant constructs were employed: Src-ΔSH2 and Src-ΔSH3 lacking the SH2-domain or SH3-domain, respectively; Src-W118K, with a functionally defective SH3-domain; Src-R175L, with a functionally defective SH2-domain; and double mutant Src- R175L/W118K with both SH2- and SH3-domains inactivated [32] [33]. SYF cells transiently transfected with Src-WT, Src-KD or the various Src mutant constructs noted above demonstrated differences in Pyk2 and FAK phosphorylation upon attachment to fibronectin-coated dishes.

**Fig 2:**
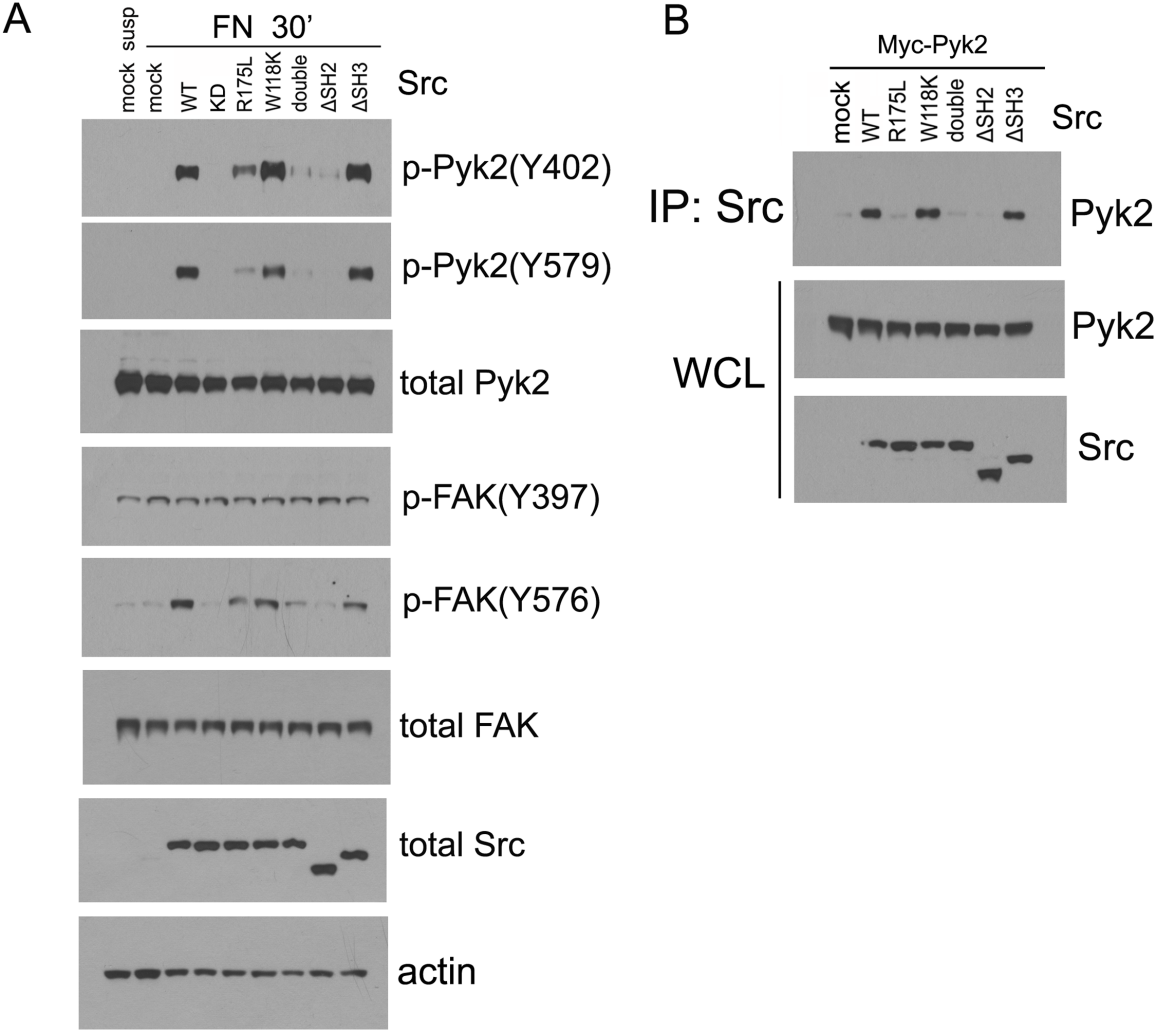
Analysis of proteins domains of Src involved in Pyk2 phosphorylation. A, SYF cells transiently transfected with Src-WT or the various Src mutant constructs were serum-starved, detached and kept in suspension in DMEM containing 0.5% BSA for 1 hr. Cells were then seeded on fibronectin-coated dishes, and allowed to attach for 30 min. Phosphorylation of Pyk2 and FAK was examined by immunoblot. B, Co-IP of Src and Pyk2. SYF cells were transiently transfected with Myc-Pyk2 along with Src-WT or Src mutant constructs, the expressed Src was immunoprecipitated and the associated Pyk2 was examined by immunoblot.

As could be predicted, FAK-Y397 phosphorylation was not affected by expression of any of these Src constructs in SYF cells upon cell attachment on fibronectin (Fig 2A). In contrast, differences were observed in the capability of the various Src constructs to mediate phosphorylation of Pyk2-Y402, Pyk2-Y579 and FAK-Y576. Src-WT readily promoted phosphorylation of these sites while Src-KD failed to do so. Src-W118K and Src-ΔSH3 constructs induced phosphorylation of these sites to the same extent as Src-WT. Src-R175L showed a much weaker effect on Pyk2-Y402, Pyk2-Y579 and FAK-Y576 phosphorylation, while Src-ΔSH2 and the double mutant Src-Rl75L/W118K failed to promote phosphorylation of these sites (Fig 2A). These data suggest that the Src SH2-domain is necessary for Src-mediated phosphorylation of Pyk2 at sites Y402 and Y579 (and of the FAK-Y576 site). Co-immunoprecipitation results were consistent with these findings. That is, in SYF cells that had been co-transfected with Myc-Pyk2-WT and the various Src constructs, Pyk2-WT readily co-precipitated with Src-WT, Src-W118K and Src-ΔSH3, but not with Src-R175L, Src-W118K/R175L or Src-ΔSH2 (Fig 2B).

### Initial adhesion-induced phosphorylation of Pyk2 by Src is independent of Pyk2 kinase activity

**Fig 3:**
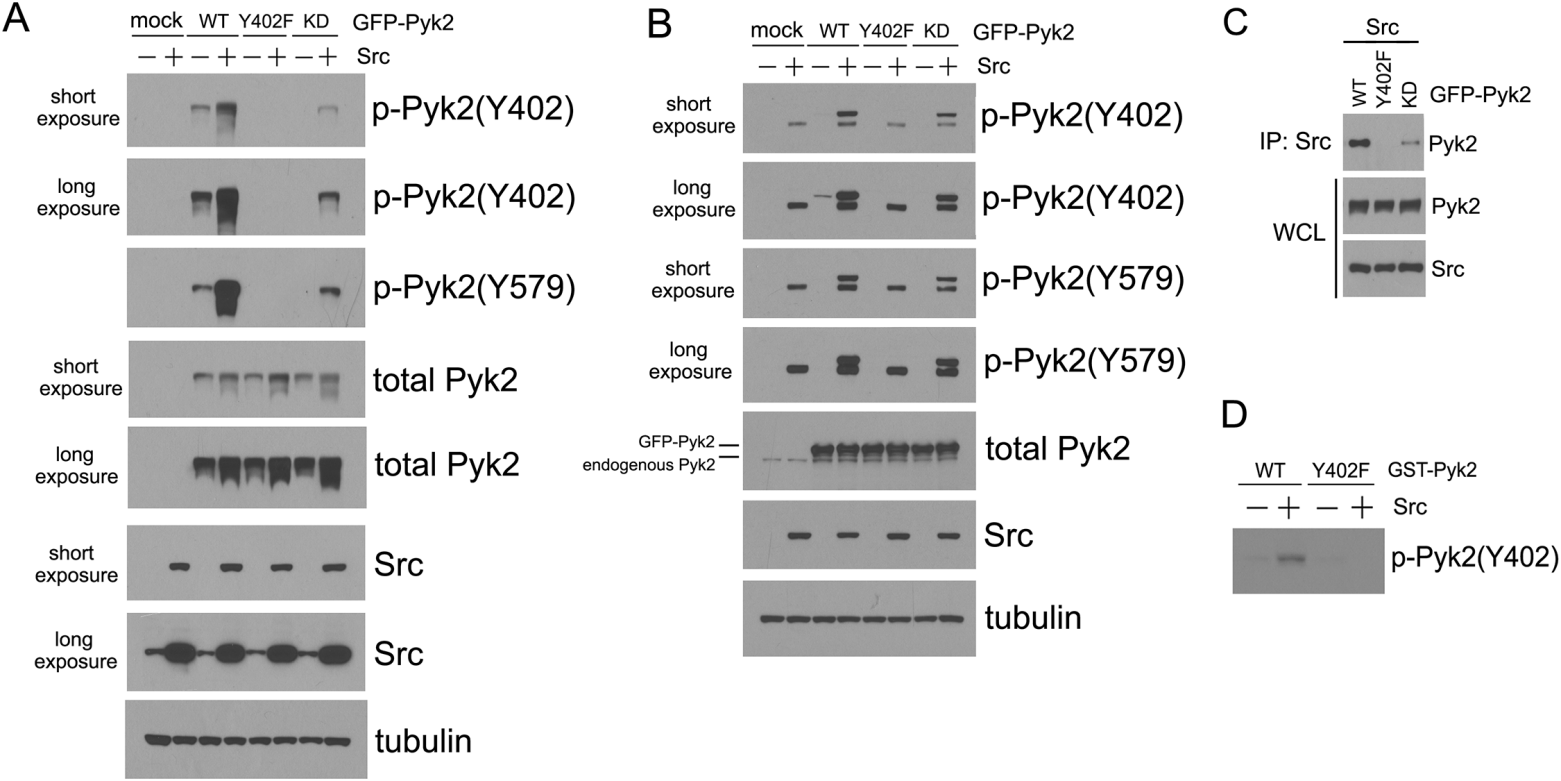
Src is required to initiate adhesion-induced Pyk2 phosphorylation. A, 293T cells were transfected with GFP-tagged Pyk2-WT, Pyk2-Y402F or Pyk2-KD, with or without Src, serum-starved, detached, and then reattached to fibronectin-coated dishes for 30 min. Cell lysates were collected and assayed by immunoblot to examine Pyk2 phosphorylation. B, SYF cells were transfected and assayed similarly as in A. C, SYF cells were co-transfected with Src and GFP-tagged Pyk2-WT, or Pyk2-Y402F, or Pyk2-KD as indicated. The expressed Src was immunoprecipitated, and the associated Pyk2 was examined by immunoblot. D, GST-Pyk2-WT and GST-Pyk2-Y402F were expressed in *E*. *coli*, purified, and employed in an in vitro kinase assay as described in Materials and Methods. Pyk2 phosphorylation by recombinant Src was examined by immunoblot analysis by using anti-phospho-Pyk2 (Y402) antibody.

Conceivably, Src may mediate Pyk2 phosphorylation upon cell adhesion by two distinct mechanisms. That is, Src may directly phosphorylate Pyk2 on Y402, or Src may activate Pyk2, resulting in Pyk2 autophosphorylation. To discern between these two possibilities, we decided to employ 293T cells as an additional model system. It has been reported previously that these cells do not express detectable levels of endogenous Pyk2 [21], and we observed the same here (Fig 3A). Thus, in these cells, we expect to see low, if any, levels of exogenously expressed Pyk2 phosphorylation due to endogenous Pyk2 potentially phosphorylating the exogenously expressed Pyk2 molecule *in trans* [21].

When expressed in 293T cells in the absence of Src, wild-type Pyk2 (Pyk2-WT) demonstrated low, although detectable levels of phosphorylation at sites Y402 and Y579 upon cell attachment on fibronectin (Fig 3A). We surmise that this is due to expression of endogenous Src in these cells at detectable levels (Fig 3A). Similar to what we observed in HeLa cells (Fig 1C), co-expression of Src resulted in an enhanced phosphorylation of Pyk2 on both Y402 and Y579 sites. When expressed alone, kinase-dead Pyk2 (Pyk2-KD) was not phosphorylated on either of the two sites upon cell adhesion. Importantly, co-expression of Src resulted in an increase in phosphorylation of Pyk2-KD on both Y402 and Y579 sites, albeit at lower levels compared to Pyk2-WT (~18-fold difference as measured by densitometry) (Fig 3A, lanes 4 and 8). In support of this notion, and in accordance with a previous report [34], our *in vitro* results show that Src can directly phosphorylate Pyk2 on Y402 (Fig 3D). Taken together, these results suggest that Src can directly phosphorylate Pyk2 in a manner that is independent of Pyk2 kinase activity, but without Pyk2 activity Src-initiated Pyk2 phosphorylation is dramatically reduced. Thus, maximal phosphorylation of Pyk2 in adherent cells in the presence of Src requires Pyk2 kinase activity.

We also examined the role of the Y402 site in Pyk2 phosphorylation by Src. When Pyk2-Y402F mutant was expressed in 293T cells with or without Src, this form of Pyk2 was not phosphorylated at the Y402 site, as expected due to the mutation (Fig 3A). Importantly and interestingly, no phosphorylation at the Y579 site was observed either, regardless of Src co-expression (Fig 3A). Thus, phosphorylation of the Y402 site by Src appears to be critical for further phosphorylation of Pyk2 at the Y579 site by Src.

Similar results were observed in the Src/Yes/Fyn-deficient SYF cells. In these cells, we expressed a GFP-tagged form of Pyk2 (with a relative molecular weight of ~145 kDa) so as to differentiate it from the endogenous Pyk2 (with a relative molecular weight of ~116 kDa) in immunoblots. When expressed in SYF cells, phosphorylation of the GFP-tagged Pyk2-WT (faint band on overexposure) or Pyk2-KD could not be detected upon cell attachment on fibronectin (Fig 3B). However, when Src was co-expressed, both Pyk2-WT and Pyk2-KD were readily phosphorylated at sites Y402 and Y579 (Fig 3B, lanes 4 and 8), further demonstrating that Src activity is indispensible for Pyk2 phosphorylation. As was observed in 293T cells, Pyk2-KD was phosphorylated at lower levels compared to Pyk2-WT upon Src co-expression, again suggesting that full phosphorylation of Pyk2, while dependent on Src, does require Pyk2 kinase activity. Consistently, no phosphorylation could be detected with Y402F mutant, confirming that Y402 phosphorylation is a prerequisite for the subsequent phosphorylation of Y579 site by Src. Consistent with these results, and with findings by others [35], we observed that the Y402 site is required for Src-Pyk2 interaction. Thus, in a co-immunoprecipitation experiment, Pyk2-WT and, to a lesser extent, Pyk2-KD, associated with co-expressed Src in SYF cells. In contrast, Pyk2-Y402F failed to do so (Fig 3C). As a corollary of these findings, we conclude that the level of Src-Pyk2 interaction seems to correlate with the level of tyrosine phosphorylation at the Y402 site of Pyk2. Taken together with the results in Fig 2, an interaction between the SH2-domain of Src and the phosphorylated Y402 site in Pyk2 appears to be crucial for the Src-Pyk2 interaction and subsequent further phosphorylation of Pyk2.

### Pyk2 autophosphorylation *in trans* is independent of Src activity

**Fig 4:**
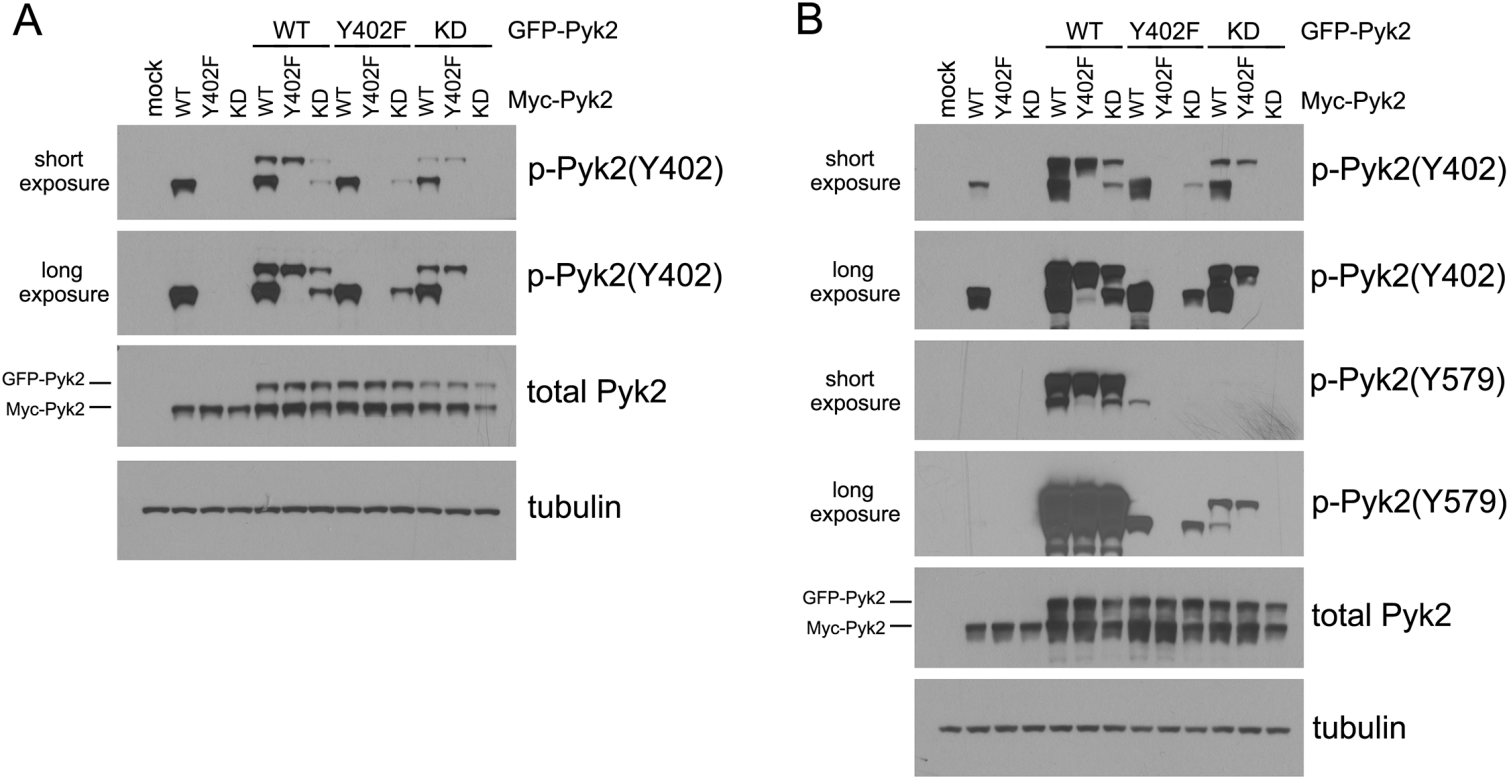
Pyk2 transactivation is independent on Src activity. SYF cells (A) and 293T cells (B) were cultured in the presence of serum and transfected with Myc-tagged Pyk2 constructs and GFP-tagged Pyk2 constructs as indicated. Pyk2 phosphorylation was detected by immunoblot. The expression of GFP-Pyk2 and Myc-Pyk2 was also studied by immunoblot of Pyk2.

Previous studies by others have suggested that Pyk2 autophosphorylates itself *in trans* at the site Y402, and that this transphosphorylation is independent of Src kinase activity[21]. Discerning from the literature, these studies have been carried out in cells that were transfected to overexpress Pyk2 and that were cultured in the presence of 10% FCS (in our experiments above, cells were serum-starved, kept in suspension and then plated on fibronectin, in order to study integrin-mediated signal transduction without the confounding effect of growth factors present in serum). The experimental conditions described by Park *et al*. were used here next. When overexpressed in SYF cells in the presence of serum, a phosphorylated form of Myc-tagged Pyk2-WT, but not of Pyk2-Y402F or Pyk2-KD mutants, could be detected (Fig 4A, first four lanes), suggesting for autophosphorylation of Pyk2 in the absence of Src. This phosphorylation was observed at a low level, presumably due to the absence of Src but enabled by significant Pyk2 overexpression (and the presence of serum). Indeed, simultaneous co-expression of Src significantly induced Pyk2 phosphorylation in these conditions (S1 Fig).

In an experiment with overexpression of various combinations of GFP-tagged-Pyk2 and Myc-tagged Pyk2 in SYF cells, Myc-Pyk2-WT phosphorylated co-expressed GFP-Pyk2-KD at Y402 via transphosphorylation (Fig 4A, lane 11), and vice versa, GFP-tagged Pyk2-WT phosphorylated Myc-tagged Pyk2-KD (lane 7). Interestingly, expression of Pyk2-KD significantly suppressed the trans-phosphorylation of co-expressed Pyk2-WT, presumably in a dominant-negative manner due to its lack of kinase activity (compare GFP-Pyk2-WT phosphorylation in lanes 5 and 7, and Myc-Pyk2-WT phosphorylation in lanes 5 and 11). Pyk2-Y402F retained kinase activity and thus didn’t affect the trans-phosphorylation of co-expressed Pyk2-WT (compare GFP-Pyk2-WT phosphorylation in lanes 5 and 6, and Myc-Pyk2-WT phosphorylation in lanes 5 and 8). Moreover, Pyk2-Y402F was found to be able to trans-phosphorylate co-expressed Pyk2-KD (lanes 10 and 12). Pyk2-KD could not get phosphorylated (lane 13) since no Pyk2 transphosphorylation or Src phosphorylation of Pyk2 occurred in these cells. Also, no (or very little) phosphorylation on Y579 of Pyk2 could be detected in any combination, presumably due to the lack of Src kinase in SYF cells. Indeed, co-expression of Src in the SYF cells was required for Y579 phosphorylation (see S1 Fig). Similar results were observed in 293T cells, except that in the presence of endogenous Src in 293T cells, phosphorylation on Y579 could be detected wherever Y402 phosphorylation occurred, further confirming that Y402 phosphorylation is indispensable for subsequent Y579 phosphorylation by Src (Fig 4B).

## Discussion

**Fig 5:**
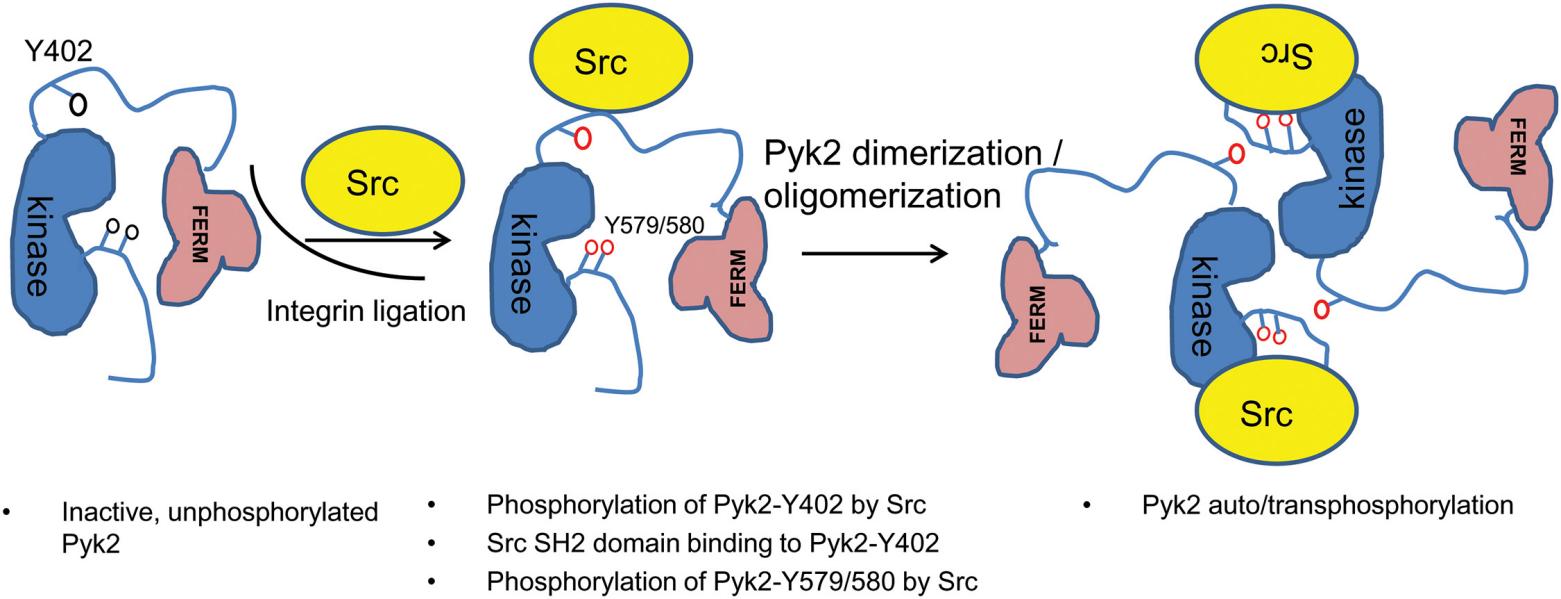
Schematic representation of Pyk2 phosphorylation triggered by Src. In the inactive state, Pyk2 is in an unphosphorylated state. Upon integrin ligand binding, Src phosphorylates Y402, creating a binding site for the SH2-domain of Src, and enabling further phosphorylation of Pyk2 by Src, including at site Y579 (and likely at Y580, although not studied here). As shown by others [21,22] phosphorylated and activated Pyk2 dimerizes or oligomerizes with itself, leading to further phosphorylation of Pyk2 via auto/trans-phosphorylation.

FAK and Pyk2 are important protein tyrosine kinases in diverse cellular events and disease processes [36]. Despite high homology, these two FAK family members appear to be activated via distinct mechanisms. In the present study, we reveal a novel role for Src in triggering Pyk2 phosphorylation (but not FAK phosphorylation) upon cell adhesion on fibronectin, followed by Pyk2 transphosphorylation and full activation (depicted schematically in Fig 5).

Like many protein kinases, FAK and Pyk2 undergo autophosphorylation upon activation. While intramolecular autophosphorylation of FAK has been confirmed by crystal structures [19], full Pyk2 activation is suggested to occur via trans-phosphorylation [21]. Src and FAK family kinases are often mutually connected and involved in many signaling events and they associate with and activate each other. Although FAK autophosphorylation is not dependent on Src, autophosphorylated FAK recruits and activates Src [15], and the associated Src can then phosphorylate FAK’s activation loop to maximize FAK kinase activity [17]. Additionally, the FAK crystal structure study suggests that once the activation loop of FAK has been phosphorylated by Src, FAK is no longer subject to inhibition by its FERM domain [19].

Our study on the role of Src in fibronectin-induced Pyk2 phosphorylation reveals another facet. Specifically, during fibronectin-mediated cell attachment, Pyk2 phosphorylation on Y402 fails to take place in the absence of Src family kinases whilst FAK autophosphorylation on Y397 is consistently detected (Figs 1D and 3B). Our results further suggest that Src exerts its effect by directly phosphorylating the Y402 site in Pyk2, as opposed to by inducing Pyk2 autophosphorylation (Fig 3). Furthermore, we found that phosphorylated Y402 site in Pyk2 forms an effective binding site for Src-Pyk2 interaction, as mutation in or deletion of the SH2-domain in Src, and mutation of the Y402 site on Pyk2, abolished Pyk2 interaction and phosphorylation by Src (Fig 2).

Our studies suggest that Src phosphorylation of Pyk2 at Y402 is an early event in Pyk2 activation by integrin signaling. As noted in the Introduction, the displacement of the FERM domain to release an auto-inhibitory interaction has been suggested to be the first step in the initiation of Pyk2 kinase activation [22,23], and our results make it plausible that the modulator eliciting such conformational change could be Src. Although the Pyk2 FERM domain plays an important role in regulating its kinase activity, the underlying mechanism for this regulation is unclear as the Pyk2 FERM domain does not bind to the Pyk2 kinase domain as is observed in the case of FAK [20]. An autonomously expressed Pyk2 FERM domain has been reported to inhibit Pyk2 phosphorylation [23,37], and in our unpublished studies, we decided to examine whether Src could alleviate this inhibitory effect. We found that while Src could, to an extent, rescue FERM inhibition of Pyk2 phosphorylation, it had no effect on FERM association with Pyk2. At present time, therefore, it doesn't seem that Src’s primary function is to displace FERM.

Our studies don’t rule out the possibility that Src may affect Y402 phosphorylation of Pyk2 through indirect means, as well. For example, Ca2+ is involved in Pyk2 homodimer formation and transphosphorylation [23], and previously Pyk2 and Src have been linked to Ca2+ signaling [38]. Indeed, depletion of intracellular Ca2+ by using Ca2+ chelator BAPTA significantly reduces fibronectin-induced Pyk2 phosphorylation (data not shown), but whether this effect is Src-dependent or -independent remains to be determined.

Our studies further indicate that phosphorylation of the Y402 site by Src is critical for further phosphorylation of Pyk2 at the Y579 site by Src (Fig 3). In keeping with the established model for FAK activation (see above), we envision that phosphorylation of Pyk2 at the Y579 site results in full activation of Pyk2. Indeed, our results suggest that full phosphorylation of Pyk2, while initially dependent on Src, requires Pyk2 kinase activity. Thus, we surmise that Src has an important “priming” role in Pyk2 phosphorylation upon cell adhesion on fibronectin. In this “priming” model, we envision an initial (weak) interaction (not detectable by co-immunoprecipitation) brings Src and an unphosphorylated Pyk2 molecule together, for example, via the SH3-domain of Src binding to a proline-rich region in Pyk2. Active Src then phosphorylates Pyk2 at Y402 that constitute a binding site for the SH2-domain Src. SH2-domain engagement then stabilizes Src-Pyk2 interaction (which is readily detectable in standard co-immunoprecipitation conditions), enabling further phosphorylation of Pyk2 by Src at Y579 (and possible at Y580 although not studied here). This activation loop phosphorylation in Pyk2 then results in Pyk2 activation that in turn results in further (full) phosphorylation of Pyk2 by itself via transphosphorylation (Fig 5). Notably, the process of initial Pyk2 phosphorylation by Src we envision here is similar to how Src phosphorylates the so-called Cas-SD domain in the docking protein Cas [39].

Collectively, our studies reveal a novel function of Src in priming Pyk2 (but not FAK) phosphorylation and subsequent activation, and shed light on the signaling events that regulate the function of Pyk2. These insights may enable further pharmaceutical development of compounds that selectively modulate FAK and Pyk2 kinases in various therapeutic applications.

## Supporting Information

S1 Fig Src induces Pyk2 phosphorylation in SYF cells. SYF cells were transfected with Pyk2 constructs in the presence or absence of Src-WT. The expression and phosphorylation of Pyk2 was examined by immunoblot.
(TIF)

## Abbreviations

FAK: focal adhesion kinase
FAT-domain: focal adhesion targeting-domain
FERM-domain: Four point-1, ezrin, radixin, and moesin-domain
MEF: mouse embryonic fibroblast
PLL: poly-l-lysine
Pyk2: proline-rich tyrosine kinase 2
